# Tripeptide IRW Protects MC3T3-E1 Cells against Ang II Stress in an AT2R Dependent Manner

**DOI:** 10.3390/molecules27123684

**Published:** 2022-06-08

**Authors:** Nan Shang, Khushwant S. Bhullar, Jianping Wu

**Affiliations:** 1 Department of Agricultural Food and Nutritional Science, University of Alberta, Edmonton, AB T6G 2R3, Canada; nshang@cau.edu.cn (N.S.); bhullar@ualberta.ca (K.S.B.); 2 College of Engineering, China Agricultural University, Beijing 100083, China

**Keywords:** osteoporosis, IRW, peptides, RUNX2, RAAS, AT2R

## Abstract

Multiple strategies including the use of bioactive peptides and other nutraceuticals are being adopted to maintain bone health. This study provides an improved and deeper understanding of the pharmacological effects that a bioactive peptide IRW (Ile-Arg-Trp) extends on bone health. Our results showed that IRW treatment protects osteoblasts against Ang II induced decline in cell proliferation and restores protein levels of collagen type I alpha 2 chain (COL1A2) and alkaline phosphatase (ALP) levels in MC3T3-E1 cells (*p* < 0.05). Apart from augmentation of these mineralization factors, the angiotensin II (Ang II) induced apoptotic stress in osteoblasts was mitigated by IRW as well. At the molecular level, IRW abolished the cytochrome-c release via modulation of pro-and anti-apoptotic genes in MC3T3-E1 cells (*p* < 0.05). Interestingly, IRW also increased cellular levels of cytoprotective local RAAS factors such as MasR, Ang (1–7), ACE2, and AT2R, and lowered the levels of Ang II effector receptor (AT1R). Further, our results indicated a lower content of inflammation and osteoclastogenesis biomarkers such as cyclooxygenase 2 (COX2), nuclear factor kappa B (NF-κB), and receptor activator of nuclear factor kappa-B ligand (RANKL) following IRW treatment in MC3T3-E1 cells (*p* < 0.05). The use of an antagonist-guided cell study indicated that IRW contributed to the process of cytoprotection and proliferation of osteoblasts via Runt-related transcription factor 2 (RUNX2) in face of Ang II stress in an AT2R dependent manner. The key findings of our study showed that IRW could potentially have a therapeutic role in the treatment and/or prevention of bone disorders.

## 1. Introduction

Bone is a living mineralized connective tissue that serves important metabolic and mechanical functions, such as supporting body structure, protecting the internal organs, storing minerals, and also as a vital endocrine organ [1]. Bone structure and integrity are maintained via homeostasis between bone-resorbing cells (osteoclasts) and bone-forming cells (osteoblasts) referred to as “coupling” [2]. Any disruption in balanced coupling can trigger a decline in bone health and subsequently the quality of life [2,3]. Among the plethora of bone disorders, metabolic bone disorder is a common pathological mechanism characterized by reduced bone mass and increased risk of fractures [3]. Among several physiological mechanisms that underline bone disorders, the renin angiotensin aldosterone system (RAAS) is critical. The RAAS dysfunction in bone has been reported to play an important role in the pathogenesis and progression of metabolic bone disorder [4].

The RAAS is a key regulator of blood pressure, fluid-electrolyte balance, and vascular resistance [5]. Apart from the systemic RAAS, local RAAS also plays vital pathophysiological functions in different organs including the brain, eye, fat tissue, and bone [6,7,8]. Dysfunctional RAAS in bone tissue plays an essential role in the pathogenesis and progression of bone disorders and loss of bone mineral density (BMD) [4]. In both cell and animal studies, local activation of RAAS increases bone resorption while its inhibition is associated with increased bone strength [9,10]. Among the local RAAS factors, angiotensin II (Ang II) is a vital factor affecting the pathological process of bone resorption and BMD loss. Ang II, the main RAAS effector, has a wide scope of action, and thus abnormalities in its functioning have many consequences on bone health [9]. Excessive activation of Ang II leads to bone resorption and is accompanied by stimulation of inflammatory mediators in bone [11,12]. Multiple studies have also reported that Ang II significantly inhibits osteoblastic activity and osteogenesis via interdependent mechanisms [9,13]. Further, an increase in local RAAS factors in bone tissues of the aging mice indicates that angiotensin II also plays a major role in age-related osteoporosis [14]. Apart from action on bone resorption, Ang II is also related to pathological oxidative stress and inflammation surge. Ang II also stimulates apoptosis of osteoblasts and augments cellular stress pathways (NF-κB and COX-2) [15,16]. Therefore, the therapeutic modulation of RAAS components such as angiotensin-converting enzyme (ACE), angiotensin type 1 receptor (AT1R), and angiotensin type 2 receptor (AT2R) play a quintessential role in fracture healing in vivo [17]. Thus, several studies have been performed to evaluate the beneficial effect of some RAAS-targeting drug candidates on the quality of bone.

Therapeutics that inhibit the overactive local RAAS pathway, namely, angiotensin-converting enzyme inhibitors (ACEi) and angiotensin receptor blockers (ARBs) are gaining increasing attention as a strategy to treat metabolic bone disorders such as osteoporosis [2]. Clinical evidence now confirms that the use of aliskiren (renin inhibitor), fosinopril (ACEi), and Olmesartan (ARB) has been shown to lower plasma Ang II and prevent BMD loss in humans [18,19,20]. However, the long-term use of these medications has been associated with serious off-target effects [21]. Therefore, natural and safe treatment and prevention options for bone diseases such as the use of bioactive peptides are gaining attention [22,23]. Bioactive peptides usually contain 2–20 amino acids with the ability to exhibit pharmacological action at the tissue level [24]. One of the pharmacologically active bioactive peptides gaining attention for its therapeutic activities is tripeptide IRW (Ile-Arg-Trp), derived from egg white ovotransferrin [25]. Chemically, IRW is composed of three amino acids (isoleucine–arginine–tryptophan); and has molecular weight of 473.57 g/mol, and isoelectric point of 11.12. In our previous study, IRW has been identified as a novel ACE inhibitory peptide and an ACE2 activator that can reduce vascular inflammation and oxidative stress [26,27]. More importantly, in our recent study, tripeptide IRW has also been found to exert osteogenic activity and could indirectly regulate osteoclastogenesis by reducing the expression of RANKL in osteoblasts [28]. Therefore, it is rational to hypothesize that tripeptide IRW has the potential to prevent osteoporosis via the modulation of RAAS for bone health management. In this present study, we investigated the effects of tripeptide IRW on Ang II-interfered osteogenesis and cellular stress in MC3T3-E1 cells.

## 2. Materials and Methods

### 2.1. Materials and Antibodies

Tripeptide IRW was synthesized with 99.9% purity validated by HPLC-MS/MS by Genscript (Piscataway, NJ, USA). Angiotensin II, dithiothreitol (DTT), Triton X-100, and alkaline phosphatase activity fluorometric assay kit were purchased from Sigma-Aldrich (St. Louis, MO, USA). Cell growth media α-MEM (A10490), fetal bovine serum (FBS), and Pen-Strep solution were purchased from Gibco/Invitrogen (Carlsbad, CA, USA). Dihydrethidium (DHE) and Hoechst 33342 were purchased from Thermo Fisher Scientific (Thermo Fisher Scientific, Burlington, ON, Canada). Annexin V-FITC apoptosis staining kit was purchased from Abcam (Cambridge, MA, USA). Rabbit monoclonal primary antibodies against AT1R, AT2R, MasR, OPG, NFκB, and ALP were obtained from Santa Cruz Biotechnology Inc. (Santa Cruz, CA, USA). Rabbit monoclonal primary antibodies against COL1A2, COX2, AT2R, RUNX2, ACE, and ACE2 were bought from Abcam (Cambridge, MA, USA). The RANKL and GAPDH were purchased from Cell signaling technology (Danvers, MA, USA). Goat anti-rabbit IRDye 680RD secondary antibody and Donkey anti-mouse 800CW secondary antibody was purchased from Licor Biosciences (Lincoln, NE, USA). All the remaining supplies used in this study were obtained from Sigma-Aldrich (St. Louis, MO, USA).

### 2.2. Cell Culture

The cell culture was performed according to our previous report [28]. Briefly, the murine osteoblastic cell line MC3T3-E1 (subclone 4, ATCC CRL-2593) was purchased from ATCC (Manassas, VA, USA). The osteoblasts were cultured in α-MEM supplemented with 10% FBS and penicillin-streptomycin in an incubator under 95% air and 5% CO_2_. The cells were sub-cultured using 0.25% trypsin every 2 to 3 days. All the experiments were performed on 70–80% confluent cells grown in tissue culture grade plates. Then, the cells were treated with IRW (50 μM and 25 μM) with/without 1 μM of Ang II for BrdU incorporation, superoxide detection, Western blotting, qPCR, and other assays.

### 2.3. Western Blot Analysis

The cells were seeded on 48 well tissue culture plates at a concentration of 1 × 10^4^ cells/well and incubated in α-MEM with 10% FBS. The cells were treated with IRW (50 μM and 25 μM) and 1 μM of Ang II. After incubation, the culture medium was removed, and the cells lysed in boiling Laemmle’s buffer containing 50 μM dithiothreitol (DTT) and 0.2% Triton-X-100 to prepare samples for Western blot as described previously. These cell lysates were run in SDS-PAGE, blotted to nitrocellulose membranes, and immunoblotted with specific antibodies. The protein bands were detected by a Licor Odyssey BioImager and quantified by densitometry using corresponding software (Licor Biosciences, Lincoln, NB, USA). Each band was normalized to its corresponding band of loading control. Cell lysates from untreated cells were loaded onto every gel. The results were expressed as a percentage of the corresponding untreated control.

### 2.4. Brdu Incorporation Assay

BrdU Incorporation assay was conducted to identify proliferating osteoblasts using BrdU (Bromodeoxyuridine/5-bromo-2′-deoxyuridine), an analog of the nucleoside thymidine. The cells were seeded on 48 well tissue culture plates at a concentration of 1 × 10^4^ cells/well and incubated in α-MEM supplemented with 10% FBS. After 4 h of incubation, the cells were treated with IRW (50 μM and 25 μM) with/without 1 μM of Ang II. Following 24 h of incubation, the cells were washed twice with PBS and fresh α-MEM with 1% FBS, containing 1% BrDU for 1 h was added. The cells were then fixed in 70% ethanol for 20 min, treated with 1N hydrochloric acid (HCl) for 20 min to antigen exposure, then permeabilized with 0.1% Triton-X-100 in phosphate buffered saline for 5 min, and blocked in 1% bovine serum albumin (BSA) in phosphate buffered saline for 60 min, and finally incubated with mouse monoclonal antibody against BrDU (1:1000) at 4 °C. All the steps except the addition of primary antibody were performed at room temperature. Following overnight incubation with the primary antibody, the cells were treated with an anti-mouse secondary antibody for 30 min in the dark. Nuclei were stained with the Hoechst 33342 nuclear dye and cells were visualized under an Olympus IX81 fluorescent microscope. For each data point, 3 random fields were chosen. The percentage of nuclei positive for BrDU staining was noted in each field and the mean was calculated.

### 2.5. Mineralization Assay

The mineralization studies were performed as described in our previous study [28]. Briefly, the degree of mineralization was determined in the 12-well plates using Alizarin Red staining. The cultured cells were incubated with IRW (50 μM and 25 μM) and 1 μM of Ang II. The medium was removed, and cells were rinsed twice with PBS. Thereafter, the cells were fixed with ice-cold 70% (*v*/*v*) ethanol for 1 h. The ethanol was removed by aspiration and cells were washed twice with Milli-Q water. The cells were then stained with 1% (*w*/*v*) Alizarin-S Red in Milli-Q (pH 4.2) for 10 min at room temperature. After washing with Milli-Q water, the samples were observed under light, and pictures were taken.

### 2.6. ALP Activity Assay

The alkaline phosphatase (ALP) activity was evaluated using the ALP assay kit according to the manufacturer’s instructions. After completion of the experimental procedure, the ALP levels were directly measured at OD 405 nm using a SpectraMax 340 plate reader (Molecular Devices, San Jose, CA, USA).

### 2.7. Superoxide Detection

Cellular superoxide generation was detected by DHE staining according to our previous study [28]. Briefly, MC3T3-E1 cells were incubated with 50 μM and 25 μM of IRW for 2 h, followed by treatment with Ang II (1 μM) for 30 min. Cells were then treated with 20 μM of DHE and incubated in the dark for 30 min. After washing twice with PBS, the image was taken by the Olympus IX81 fluorescent microscope, and the total fluorescence intensity was quantified by Image J software (version 1.53a; National Institutes of Health, Bethesda, MD, USA).

### 2.8. Apoptosis Assay

Cell apoptosis was measured using the Annexin V-FITC apoptosis staining kit according to the manufacturer’s instructions. The cells were seeded on 6-well tissue culture plates at a concentration of 1 × 10^4^ cells/well and incubated in α-MEM with 10% FBS until confluence. After being treated with IRW (50 μM and 25 μM) with/without 1 μM of Ang II, the adherent cells were washed once with PBS, then trypsinized, and centrifuged at 500× *g* for 5 min. The cell pellets collected were resuspended in 500 μL 1X Annexin V binding buffer, and 5 μL of Annexin V-FITC and propidium iodide was added. Then, the mixture was incubated at room temperature for 5 min in dark and analyzed by flow cytometry (Ex = 488 nm; Em = 530 nm).

### 2.9. RNA Extraction and qPCR

RNA extraction and qPCR were performed as described in our recent report [29]. The cells were cultured in 100 mm dishes and treated with IRW (50 μM and 25 μM) and 1 μM of Ang II. After incubation, the culture medium was removed, and total RNA was isolated from cells with TRIzol reagent according to the manufacturer’s instructions. cDNA was synthesized from 1 μg of total RNA using the high-capacity cDNA reverse transcriptase kit (Thermo Fisher Scientific, Burlington, ON, Canada). The primers used in the study were synthesized by Integrated DNA Technologies (IDT) with no additional modifications.

### 2.10. Statistical Analysis

All data are presented as mean ± SEM (standard error of the mean) with at least 3 independent experiments. Data were analyzed using one way analysis of variance (ANOVA) with Dunnett’s post hoc test for comparisons to control. The PRISM 6 statistical software (GraphPad Software, San Diego, CA, USA) was used for the analyses. *p* < 0.05 was considered significant.

## 3. Results

### 3.1. Impact of IRW Osteoblastic Activity against Ang II Stress in Bone Cells

**Figure 1:**
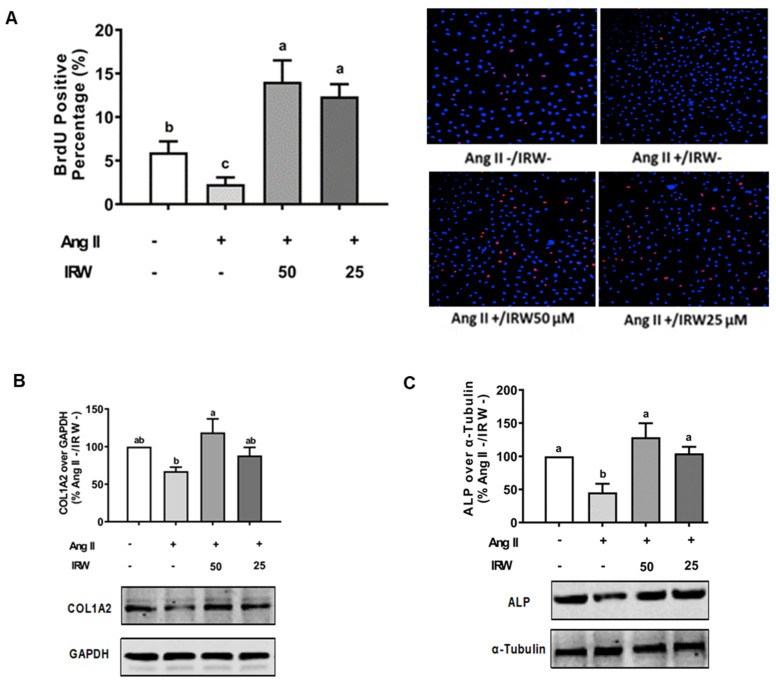

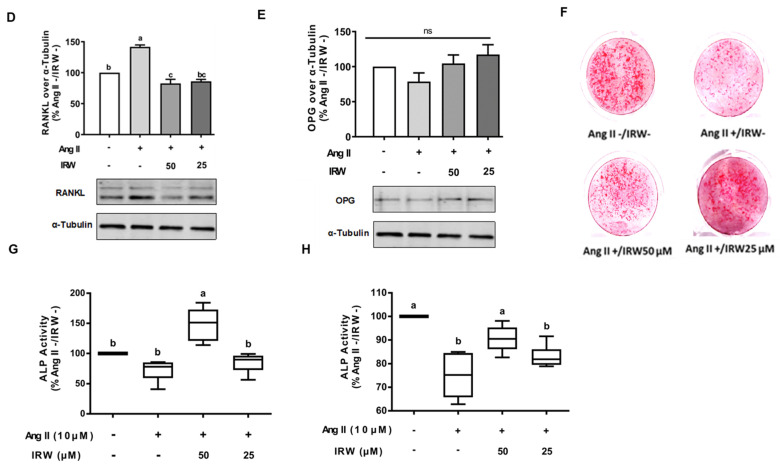
The effect of IRW on promoting osteoblastic activity against Ang II stimulation. (**A**) Osteoblast cells MC3T3-E1 were pre-treated with IRW (50 μM and 25 μM) for 2 h prior to Ang II (1 μM) and then co-cultured for 24 h. BrDU incorporation assay (**A**) was performed as described in methodology and the BrDU positive cells were counted; (**B**–**H**) osteoblast cells MC3T3-E1 were pre-treated with IRW (50 μM and 25 μM) for 2 h prior to Ang II (1 μM) and then co-cultured for 24 h. Whole cell lysates were used for the Western blotting analysis of COL1A2 (**B**), ALP (**C**), RANKL (**D**), and OPG (**E**). (**F**) Osteoblast cells MC3T3-E1 were cultured in mineralization medium (MEM-α medium with ascorbic acid and β-glycerophosphate) and treated with IRW (50 μM and 25 μM) and Ang II (1 μM) for 15 days. Alizarin Red staining was performed, and the images were captured. (**G**,**H**) Osteoblast cells MC3T3-E1 were pre-treated with IRW (50 μM and 25 μM) for 2 h prior to Ang II (1 μM) and then co-cultured for 24 h. Both the culture medium (**G**) and whole cell lysates (**H**) were collected for ALP activity analysis. All results are representative of 4~6 independent experiments and expressed as mean ± SEM. Mean without a common letter indicated *p* < 0.05.

The treatment of Ang II (1 μM) led to a significant decline in BrdU positive osteoblasts indicating an impeded cell proliferation (*p* < 0.05) (Figure 1A). Treatment with IRW (50 μM and 25 μM) successfully mitigated the cytotoxicity induced by Ang II and lead to an increase in BrdU positive cells, compared to both vehicle and Ang II groups (*p* < 0.05) (Figure 1A). Similarly, treatment with IRW (50 μM and 25 μM) successfully alleviated the Ang II-induced decline in COL1A2 and ALP expression in MC3T3-E1 cells (*p* < 0.05) (Figure 1B,C). Further, Ang II-induced increase in RANKL, a vital factor of osteoclastic bone resorption, was diminished following IRW (50 μM and 25 μM) treatment in MC3T3-E1 cells (*p* < 0.05) (Figure 1D). However, cellular levels of OPG remained unaffected by both Ang II stress and IRW treatment (Figure 1E). The osteoblast mineralization (Figure 1F) and ALP activity in both cells (Figure 1G) and media (Figure 1H) further confirmed the ability of IRW to counter Ang II-induced decrease in osteoblastic activity and trigger osteogenesis in MC3T3-E1 cells (*p* < 0.05) (Figure 1F,G).

### 3.2. Cytoprotective Role of IRW against Apoptotic Activity of Ang II in Bone Cells

**Figure 2:**
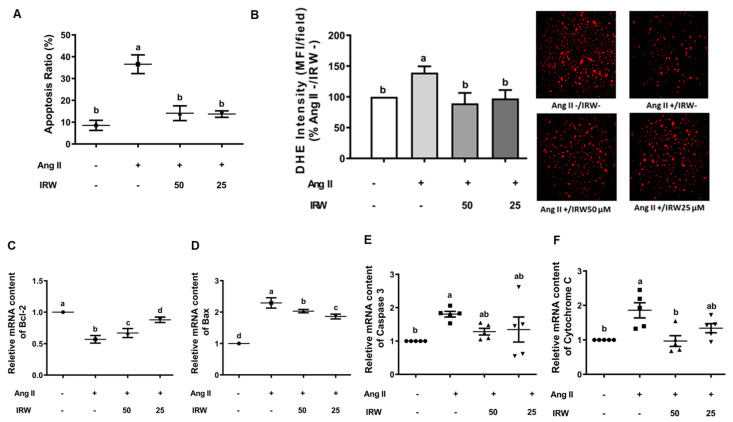
The effect of IRW on preventing osteoblast cell apoptosis against Ang II stimulation. Osteoblast cells MC3T3-E1 were pre-treated with IRW (50 μM and 25 μM) for 2 h prior to Ang II (1 μM) and then co-cultured for 24 h. (**A**) Cells were collected, fixed, and the cell apoptosis rate was measured by flow cytometry; (**B**) DHE staining was performed to measure the levels of oxidative stress; (**C**–**F**) total RNA was extracted using TRIzol, converted to cDNA, and then the gene expression of mitochondrial apoptotic markers (**C**) Bcl-2, (**D**) Bax, (**E**) Caspase 3, and (**F**) Cytochrome C was measured by qPCR (quantitative PCR). All results are representative of 4~6 independent experiments and expressed as mean ± SEM. Mean without a common letter indicated *p* < 0.05.

Ang II induces oxidative stress and apoptosis through mitochondria-dependent mechanisms [30]. Our results confirmed that Ang II (1 μM) induced apoptosis and oxidative stress in MC3T3-E1 cells as evident by flow cytometry and DHE assay (*p* < 0.05) (Figure 2A,B). Like the BrdU assay (Figure 1A), treatment with IRW (50 μM and 25 μM) successfully mitigated the apoptosis and oxidative stress compared to the Ang II stress group (*p* < 0.05) (Figure 2A,B). At the molecular level, IRW treatment (50 μM and 25 μM) modulated both pro-survival and pro-apoptotic members of the apoptosis cascade. IRW treatment (50 μM and 25 μM) significantly increased pro-survival *Bcl-2* and decreased pro-apoptotic *Bax* leading to lowering of cytochrome-c release (*p* < 0.05) in MC3T3-E1 cells (Figure 2C,D,F). However, the mRNA content of *caspase-3* was marginally altered after treatment with IRW (50 μM and 25 μM) (Figure 2E).

### 3.3. IRW Modulates RAAS Factors against Ang II Stress in Bone Cells

**Figure 3:**
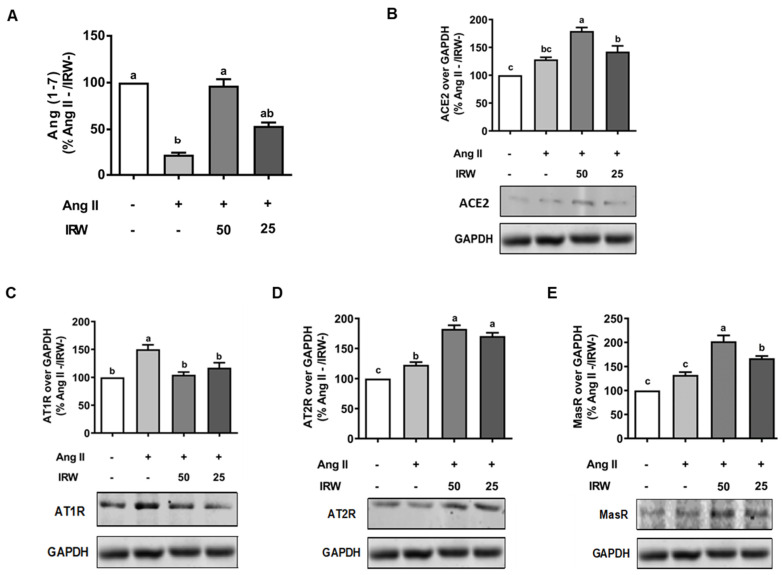
The effect of IRW on regulating RAAS signaling with Ang II stimulation. Osteoblast cells MC3T3-E1 were pre-treated with IRW (50 μM and 25 μM) for 2 h prior to Ang II (1 μM) and then co-cultured for 24 h. Whole cell lysates were collected and used for the ELISA and Western blotting analysis. (**A**) The RIPA buffer extracts were used for measuring Ang (1–7) levels using ELISA and the protein expression of (**B**) angiotensin-converting enzyme 2 (ACE II), (**C**) angiotensin II receptor type 1 (AT1R), (**D**) angiotensin II receptor type 2 (AT2R), and (**E**) Mas receptor (MasR) were measured using Western blot. All results are representative of 6 independent experiments and expressed as mean ± SEM. Mean without a common letter indicated *p* < 0.05.

Similar to cytotoxic initiation of oxidative stress and apoptosis, Ang II (1 μM) induced a decline in cytoprotective angiotensin 1–7 (Ang 1–7), while IRW treatment reversed the Ang II driven decline in Ang 1–7 in MC3T3-E1 cells (*p* < 0.05) (Figure 3A) confirming its cytoprotective nature. This was accompanied by increased ACE2 levels in MC3T3-E1 cells as well (*p* < 0.05) (Figure 3B). As both ACE2 and Ang 1–7 have emerged as key protective pathways, their increase supplements the antioxidant and anti-apoptotic properties of IRW in osteoblasts (Figure 1 and Figure 2) [27,31]. Similarly, an increase in pro-apoptotic AT1R levels by Ang II stress (1 μM) was reversed by IRW treatment (Figure 3C) [32]. Further, IRW treatment led to a significant increase in AT2R and Mas receptor (MasR) in MC3T3-E1 cells (*p* < 0.05) (Figure 3D,E). These results showed that IRW successfully modulated the pro-stress and pro-survival members of the RAAS pathway in MC3T3-E1 cells.

### 3.4. IRW Mitigates Inflammation Induced by Ang II Stress in Bone Cells

**Figure 4:**
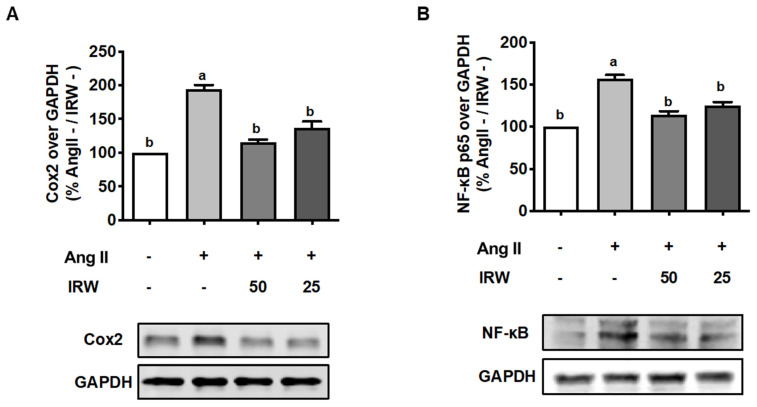
The effect of IRW on Cox2 and NF-κB p65 with Ang II stimulation. Osteoblast cells MC3T3-E1 were pre-treated with IRW (50 μM and 25 μM) for 2 h prior to Ang II (1 μM) and then co-cultured for 24 h. Whole cell lysates were collected and used for the Western blotting analysis to measure the protein expression of (**A**) cyclooxygenase-2 (Cox2) and (**B**) NF-κB p65. All results are representative of 6 independent experiments and expressed as mean ± SEM. Mean without a common letter indicated *p* < 0.05.

As inflammation skews the process of coupling towards bone resorption [33], its inhibition can play important role in the restoration of bone health. Our results showed that Ang II (1 μM) triggered a strong increase in inflammatory markers including COX2 and NF-κB (Figure 4A,B). This increase in both biomarkers was attenuated back to the basal levels following treatment with IRW (50 μM and 25 μM) in MC3T3-E1 cells (*p* < 0.05) (Figure 4A,B). These results support the earlier findings of cytoprotection by IRW in MC3T3-E1 cells (Figure 1).

### 3.5. IRW Mitigates Cellular Stress Induced by Ang II in AT2R Dependent Manner in Bone Cells

**Figure 5:**
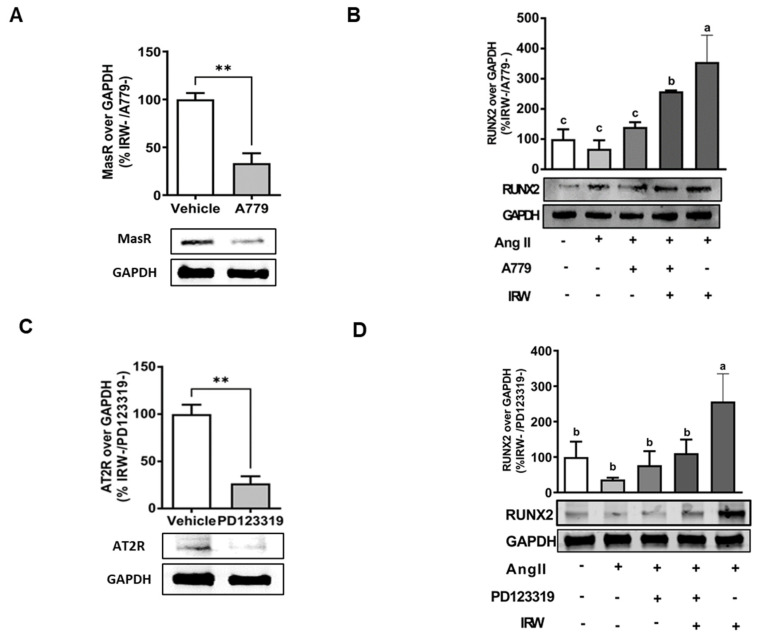
The impact of AT2R and MasR on cytoprotective activity of IRW against Ang II stimulation. Osteoblast cells MC3T3-E1 were pre-treated with IRW (50 μM and 25 μM) for 2 h prior to Ang II (1 μM) and then co-cultured for 24 h with or without AT2R (PD123319) and MasR (A779) inhibitors (**A**,**C**). Whole cell lysates were collected and used for the Western blotting analysis was conducted to measure the protein expression of (**B**,**D**) RUNX2. All results are representative of 3–6 independent experiments and expressed as mean ± SEM. Mean without a common letter indicated *p* < 0.05 while ** indicates *p* < 0.01.

In order to understand the underlying mechanisms supporting the osteogenic/cytoprotective role of IRW, the expression of vital protective RAAS receptors AT2R and MasR was inhibited using their specific inhibitions in the presence and absence of IRW (50 μM). The use of MasRi (A779, 1 μM) and AT2Ri (PD123319, 1 μM) efficiently inhibited MasR and AT2R, respectively, in MC3T3-E1 cells (*p* < 0.01) (Figure 4A,B). Next, our results showed that IRW treatment (50 μM) exhibited a significant increase in RUNX2, a vital factor for osteoblast proliferation, in presence of MasRi (A779, 1 μM). However, in presence of AT2Ri (PD123319, 1 μM), the ability of IRW to increase RUNX2 was diminished in MC3T3-E1 cells (*p* < 0.05) (Figure 5B,D).

## 4. Discussion

**Figure 6:**
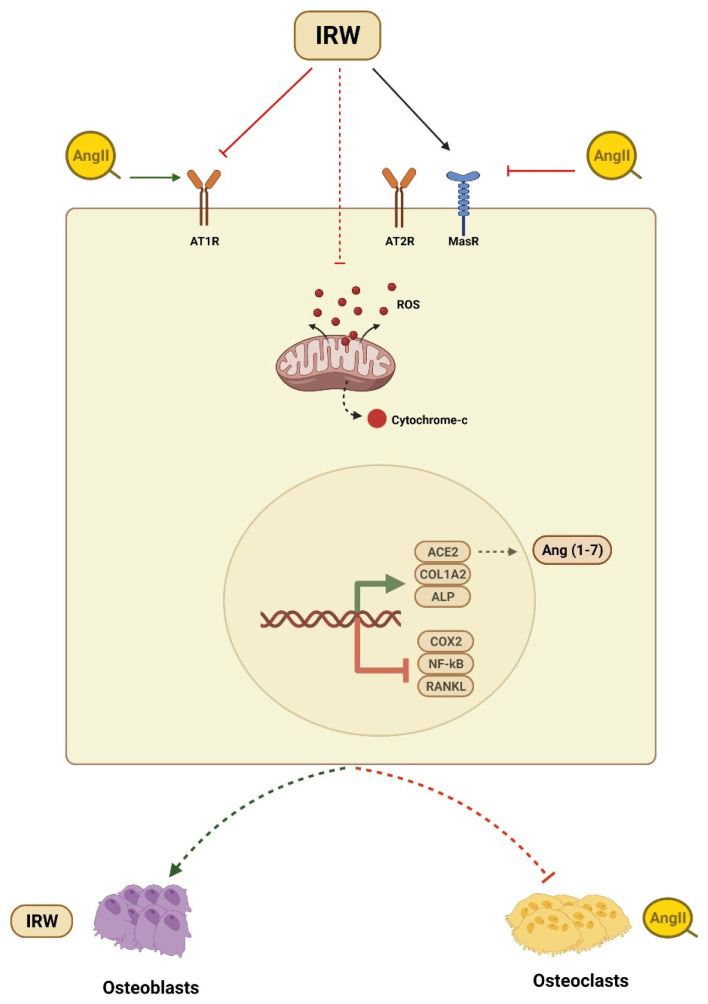
Summary of cytoprotective and osteogenic impact of IRW against Ang II stimulation.

Osteoporosis is a common bone disorder characterized by low BMD and impaired bone microstructure which leads to increased bone fragility and fracture risk [34]. Apart from being a global healthcare challenge, osteoporosis instills a major economic burden costing approximately USD 17.9 and CAD 4.6 billion per year in the USA and Canada, respectively [34,35]. Among vital factors contributing to the etiology of osteoporosis is the overactivation of the local RAAS system in bone tissues and its blockade has been shown to improve the osteoporotic incidence [36,37]. Animal studies have shown that the increased expression of Ang II-induced bone loss via RANKL-mediated osteoclasts increase [14]. Similarly, in humans, overexpressed RAAS genes such as AT1R expression and the RANKL/OPG ratio were negatively related to BMD in osteoporosis patients [38]. Different RAAS modulators can diminish osteoclasts and boost osteoblasts through AT1R, OPG/RANKL, ACE2/Ang (1–7)/Mas cascades [2,10,13,20,28,37]. Therefore, targeting the local RAAS in bone tissue is an efficient and logical strategy to counter a decline in bone health. With a global increase in aging demographics, the importance of the nutrition-mediated prevention of osteoporotic incidence is increasing tremendously. In the present study, we showed the ability of dietary peptide IRW to counter Ang II stress in osteoblasts, its mode of cytoprotection, and underlying mechanisms of action in MC3T3-E1 cells (Figure 6).

Over the past few decades, there have been rapid advancements in interventions using bioactive peptides for osteoporosis and bone health. Similar to our findings, both food-derived bioactive peptides and endogenous proteins-derived peptides have exhibited osteogenic activities. Among food derived peptides, casein phosphopeptides (CPP) were first shown to exhibit bone protecting properties in vivo [39]. Similarly, peptide rich collagen, blue mussel, and shark protein hydrolysates were shown to exhibit osteogenic effects in vivo [21,40,41]. Similar to our findings, Pro-Hyp, a collagen derived dipeptide (0.1 and 1 mM) exhibited an increase in *Runx2* and Collα1 gene expression in MC3T3-E1 cells [42]. Like IRW, low-molecular weight peptides derived from collagen also inhibited apoptosis and promote the proliferation and differentiation of MC3T3-E1 cells by activating the PI3K/Akt signaling pathway [43]. Similarly, milk derived tripeptides IPP and VPP (5 and 50 μM) exhibited an increase in osteogenesis genes in osteoblasts differentiated from human mesenchymal stem cells [44]. These results were confirmed in a follow-up study indicating that IPP (50 μM) stimulated an increase in mineralization accompanied by an increase in RUNX2 and depleted the RANKL/OPG ratio [45]. Additionally, a tripeptide KSA derived from the bone tissue of the marine fish promoted the proliferation and mineralization of MC3T3-E1 cells [46]. Our findings were also similar to tetrapeptide MPDW (37.5, 75, and 150 μM) which improved bone mineralization in both human osteoblastic cells (MG-63) and murine mesenchymal stem cells (D1) [47]. Likewise, peptides NAVPITPTL (30 ng of BSA equivalents/mL) and VLPVPQK (50 and 100 μg/kg/d for 8 weeks) were derived from buffalo milk exhibited osteogenic effects in cells and in ovariectomized rats [48,49]. Similarly, peptides derived from endogenous proteins such as KIPKASSVPTELSAISTLYL (human BMP-2; 73–92 peptide) exhibited osteogenic effects in C3H10T1/2 cells and repairs rat tibial bone defects in vivo [50]. Our findings are also similar to two peptides obtained from human BMP-7, GQGFSYPYKAVFSTQ, and VEHDKEFFHPRYHHR improved expression of osteogenic genes and enhanced bone formation in different cells [51,52]. Our results are also akin to denosumab, a key RANKL targeting drug in the treatment of osteoporosis [53]. Similar to IRW, a recently developed drug called teriparatide also exhibited an extremely efficacious osteogenesis effect [54]. It will be interesting to see if IRW supplementation in humans is tolerable and if the osteogenic effects are translated clinically as well [23]. In summary, our experiments align with multiple evidence indicating osteogenic and cytoprotective efficiency of food derived peptides and related molecules.

At the molecular level, apart from the modulation of the RAAS cascade, IRW also impacted apoptosis and inflammation pathways, both critical to the process of bone resorption. Mechanistically, caspase-3 activity plays a crucial role in osteoblast apoptosis [55]. The release of activated caspase occurs via Bcl2 family members including the apoptotic members Bad, Bax, and Bid, and anti-apoptotic Bcl-2 [56]. Disruption in normal bone remodeling is characterized by apoptosis of bone cells, especially osteoblasts, leading to diseases such as osteoporosis [57]. For example, osteoblast apoptosis occurs extensively in the proximal femur in osteoporotic subjects [58]. Therefore, anti-apoptotic processes including inhibition of caspase and modulation of Bcl-2 proteins present an effective approach. Research evidence has previously shown that increased expression of Bcl-2 prevents apoptosis of osteoblast through cytochrome C release inhibition [59]. Casp3Inh (Z-DEVD-FMK), a tetrapeptide caspase-3 inhibitor, has shown to exhibit cytoprotection in MC3T3-E1 cells and improve BMD in vivo as well [55,60,61]. Our results showed efficiency and pro-survival modulation of these key players (Bcl2, caspase-3, and cytochrome-c) in the apoptosis cascade. Similar to IRW, anti-apoptosis drugs such as alendronate, hormones, estrogens, and androgens also inhibit apoptosis of osteoblasts in cell and in vivo [61,62,63]. Similar to apoptosis, modulation of inflammatory biomarkers such as COX2, RANKL, and NF-κB by IRW supplements its overall pharmacological and osteogenic activity. These findings are in line with a previous study showing COX2 knockout leading to protection of bone tissue in vivo [64]. Similar to IRW, dexamethasone has been shown to inhibit inflammation via disruption of the NF-κB pathway [65]. Additionally, the active form of vitamin D, a major player in bone health, binds to the nuclear vitamin D receptor leading to the downregulation of NF-κB [66]. Similarly, curcumin, a vital antioxidant and cytoprotective agent inhibits RANKL induced NF-κB osteoclastogenesis in RAW 264.7 cells [67]. Taken together, IRW shows strong cytoprotective activity and boosts osteoblasts proliferation via synergistic activation of RAAS and inflammatory pathways. We also show AT2R as the pivotal receptor controlling the osteogenic activities of IRW. Ang II is a vital hormone member of the RAAS cascade which acts through AT1R and AT2R [68]. Between these two receptors, AT1R is a proinflammatory role while AT2R is involved in anti-inflammatory effects in different cell types [69,70,71]. The discovery of compound 21, an AT2R agonist, has further validated the pharmacological and anti-inflammatory role of AT2R [72]. IRW mimics the effects of AT2R agonist as it inhibits the NFκB surge triggered by Ang II in MC3T3-E1 cells [68,73]. These effects of IRW can be summed as the “counter-regulatory” to Ang II triggered AT1R stimulation. The role of AT2R in tissue repair and regeneration has been shown in both cell and animal models, thus supporting the cytoprotective and osteogenic role of IRW [74].

Our study has some limitations and research gaps. Firstly, the choice of a single cell line MC3T3-E1 cells limits the applicability of the results across species. The use of multiple cell types including Saos-2 and MG-63 would have better shown the osteogenic ability of IRW. Next, the lack of animal study limits the human applicability as a study using ovariectomized and/or hypertensive rats can better help understand the pharmacological impact of IRW on bone health in vivo. Another limitation is the lack of probing for MAPK p44/p42 pathway and STAT translocation, which are a vital downstream target of AT2R activation [74]. Additionally, a comparison of IRW with the AT2R agonist compound 21 could hence support and highlight the pharmacological potential of the tripeptide. Yet, despite these limitations, our findings confirm the cytoprotective, anti-inflammatory, and osteogenic role of IRW against Ang II stress in osteoblasts (Figure 6). However, further research is needed to clarify the impact of IRW on bone tissue using animal models of bone diseases.

## 5. Conclusions

Dietary peptide IRW is a unique pharmacological peptide with a diverse spectrum of biological activities. Among these anti-inflammatory, renin angiotensin aldosterone system (RAAS), and bone rejuvenation are of vital interest. In the current study, we showed the ability of IRW to counter angiotensin II (Ang II) stress and replenish bone synthesis factors such as RUNX2 and COL1A2 in MC3T3-E1 cells. Our results showed IRW exhibited cytoprotective activity against Ang II stress in MC3T3-E1 cells as evident by sustained and proliferating osteoblasts. This was also accompanied by a decrease in Ang II-induced oxidative stress and pro-apoptosis factors such as Bcl2, Bax, caspase 3, and cytochrome C. As the RAAS pathway plays a vital role in bone health and countering Ang II stress, our results showed an increase in levels of ACE2, AT2R, and angiotensin (1–7); and depleted levels of AT1R following IRW treatment. The pharmacological impact of IRW in bone cells was further supported by a decline in RANKL, COX2, and NF-κB. Using inhibitors of AT2R and MasR, we elucidated that the ability of IRW to stimulate bone anabolism was dependent on AT2R in MC3T3-E1 cells. However, further in vivo investigations using Ang II-focused bone health models are warranted to see in vivo translation of findings from this cell study, and IRW’s possible role(s) as therapeutic in osteoporosis and metabolic bone diseases.

## Data Availability

Data sharing is not applicable to this article.

## Sample Availability

Samples of the compounds are not available from the authors.
